# Supplementary material for: Are carbon emissions trading and green financial instruments synergistic? -Comprehensive quantitative research based on content analysis
Source: PLoS One. 2024 Mar 7;19(3):e0298601. doi: 10.1371/journal.pone.0298601 (PMC10919601; doi:10.1371/journal.pone.0298601)
Supplement: S1 Data — (ZIP) [file pone.0298601.s001.zip › Data/地级市Z_F工作报告.zip!/╒π╜¡/║╝╓▌╩╨/└┤╫╘╣1⁄2╓┌║┼í╛┬φ┐╦╩2╛▌═°í┐_2014.docx]

各位代表：

　　现在，我代表市人民政府向大会报告工作，请予审议，并请市政协委员和其他列席人员提出意见。

　　一、2013年工作回顾

　　2013年，面对复杂多变的外部环境和频发的自然灾害，在省委省政府和市委的正确领导下，全市上下以科学发展观为统领，以加快转变经济发展方式为主线，积极应对各种困难挑战，稳增长、促转型、惠民生，经济社会保持平稳健康发展。全市实现地区生产总值8343.52亿元，增长8.0%；地方财政收入945.20亿元，增长9.9%；城镇居民人均可支配收入39310元，农村居民人均纯收入18923元，分别增长10.1%和11.2%；城镇登记失业率1.85%；居民消费价格涨幅2.5%；人口自然增长率4.73‰；减排指标完成上级下达任务。

　　（一）抓投资，扩需求，经济保持平稳增长

　　有效投资持续扩大。全市实现固定资产投资4263.87亿元，增长14.5%，其中重点建设项目投资1190亿元，占27.9%。完成基础设施投资852.47亿元，增长9.5%。完成工业投资910.46亿元，增长6.9%。完成民间投资2275.21亿元，增长10.5%。新增建设用地4.78万亩，消化批而未供土地6.72万亩。消费水平稳步提升。全市实现社会消费品零售总额3531.17亿元，增长13.0%。实现网络零售额1524.12亿元，增长68.8%。首发杭州通·旅游消费卡10万张，开展促消费活动258项。武林商圈获“中国最具竞争力中央商务区”称号。国家电子商务产品监测中心落户杭州。成功举办西湖国际博览会、国际动漫节、文化博览会，荣获中国十大最具影响力会展城市、最受欢迎国际会奖旅游城市等荣誉。开放型经济稳中向好。全市实际到位外资52.76亿美元，超额完成省下达任务。引进世界500强投资项目15个，投资3000万美元以上项目131个。新批对外投资项目129个，境外投资27.27亿美元，增长2.8倍。实现市属出口384.16亿美元，增长10.4%。离岸服务外包合同执行额35.64亿美元，增长20.1%。启动国家跨境贸易电子商务服务试点工作。到位内资860.60亿元，增长10.5%，其中浙商回归到位资金480.19亿元，投资5亿元以上项目25个。加强长三角区域合作。完成援疆援藏等对口支援和山海协作任务。继续推进外事、港澳、台侨等工作。

　　（二）抓创新，重质量，产业结构不断优化

　　创新活力不断增强。加快推进国家科技金融试点城市、知识产权示范城市、质量强市示范城市建设。实施69个市重大科技创新项目，新增国家高新技术企业223家。杭州高新区位列全国高新区综合排名第六名，首次进入第一方阵。杭州经济技术开发区在全国国家级开发区综合考评中取得历史最好成绩。全市高新技术产业销售产值3895.88亿元，占规上工业销售产值比重达28.9%。规上工业新产品产值率为28.2%，创历史新高。全社会研发投入占GDP比重预计2.95%。发明专利授权量4915件，保持省会城市第一。继续实施“115”引智计划，新增“国千”人选13名、“省千”人选58名，市“521”计划人选30名、团队2个。十大产业引领发展。实施重点产业创新发展三年行动计划，十大产业实现增加值3908.74亿元，增长12.1%，占GDP比重达46.9%。三次产业比重调整为3.2:43.9:52.9。国税收入首次突破千亿元，列省会城市第二。电子商务、信息软件、文化创意、物联网产业增加值分别增长55.7%、23.5%、18.0%和15.8%。文化创意、金融服务、信息软件、旅游休闲、电子商务等成为杭州新的优势产业。建立首个“两岸文创产业合作实验区”，运河、西湖国家级广告产业园加快推进。新引进重点金融服务机构12家，境外上市企业4家，浙江股权交易中心挂牌企业14家。杭州银行设立国内首家文创金融专业支行。传统优势产业加快提升。深入实施“四换三名”工程。淘汰高污染高能耗企业230家，腾出用地2055亩，腾出用能指标46万吨标煤。实施“机器换人”重点技改项目199个，认定“机器换人”示范企业40家。完成旧厂区改造建筑面积863.65万平方米，盘活存量土地17449亩。25家企业入选中国企业500强，30家企业入选中国制造业企业500强。53家企业入选中国民营企业500强，保持全国第一。推进“两化”深度融合，加快传统优势产业改造提升。工业技改投入737.31亿元，占工业投资比重达81.0%。完成“个转企”13114家、“无转有”19789家、“小升规”1139家。现代农业稳步发展。实现农林牧渔业总产值399.76亿元，增长4.0%。十大农业主导产业产值334.08亿元，占农业总产值83.6%。深化农村综合改革，流转土地承包经营权5万亩。建设高标准农田33万亩，垦造耕地2万亩。深入推进农业“两区”建设，新建各级粮食生产功能区201个，通过省级验收现代农业园区达48个。设施农业达55万亩。新增“菜篮子”基地66个，主要农产品自给率达56.5%。新增市级以上农业龙头企业55家，新增家庭农场551家，新成立农民专业合作社640家。健全农产品质量安全体系，农业“三品”认证率达55.2%。

　　（三）抓统筹，优功能，城乡建设不断发展

　　基础设施加快建设。铁路东站枢纽投入使用，杭宁、杭甬客专开通运行。地铁2号线东南段全线贯通，1号线下沙延伸段和4号线首通段5个车站主体工程完工。地铁二期建设规划获批。整治和建设延安路、同协路、沿江大道等城市主次干道130条，打通断头路13条。建成德胜高架、彩虹大道（滨江段）等快速路和钱江通道，之江大桥投入使用。建成110千伏及以上变电容量316.6万千伏安。开发利用地下空间775万平方米。城西污水处理厂（一期）建成，闲林水库大坝主体工程完工。杭甬运河（杭州段）全线贯通。城市管理水平不断提高。开展“贴心城管”行动，推进智慧城管建设，城市“洁化、绿化、亮化、序化”水平继续提升。荣获“最佳中国形象城市”称号。强势推进城乡“三改一拆”，超额完成省下达任务。地铁1号线日均运送乘客达32万人次。新增优化公交线路49条，建成公交专用道50公里，新增公交车512辆，主城区公交分担率提高3.2个百分点。新增停车泊位59852个，开工建设公共停车泊位15253个。完善智能交通控制系统，实行路口300米机动车严管等措施，治理交通拥堵点28个。镇村建设加快推进。深入推进扩权强镇，实施中心镇“双千工程”。实施大企业大集团与中心镇合作项目19个，总投资110.3亿元。建成农村文化礼堂148个。创建中心村193个、美丽乡村精品村62个、风情小镇8个、精品线路14条、精品区块7个。全国改善农村人居环境现场会在桐庐召开。新增中心镇小贷公司6家、村镇银行2家。改造农村住房3.08万户，下山移民4692人。余杭区塘栖镇获省级小城镇试点考核第一。城乡统筹成效明显。“全面西进”步伐加快，县（市）经济实力得到提升。五县（市）GDP、固定资产投资、社会消费品零售总额、外贸出口增幅分别高于全市平均水平0.8、7.3、2.7、2.9个百分点，成为我市经济发展的新增长极。实施区县（市）协作项目126个，到位协作资金3.6亿元，落实“联乡结村”帮扶资金1.42亿元。改造提升农家小型标准超市693家，“万村千乡”工程实现全覆盖。市属医院托管县（市）级医院9家。新组建中心乡镇学校（幼儿园）互助共同体112个，覆盖率达98.6%。

　　（四）抓保障，强服务，民生事业不断提升

　　民生保障不断完善。圆满完成为民十件实事。全市城镇新增就业23.19万人，帮助城镇失业人员实现再就业13.13万人。新增大学生创业企业1381家。社会基本养老保险、基本医疗保险、失业保险参保人数分别达到637.10万人、822.28万人和316.36万人。获评全国首批电子社保示范城市。开工建设保障性住房3.6万套，竣工3.9万套。有序推动经济适用房上市交易与回购工作，实现廉租房和公共租赁房并轨运行。市第三福利院建成启用。全市新增养老床位7028张，培训专业养老护理员1963名。主城区基本实现居家“智慧养老”15分钟服务圈。继续推进农村“三位一体”养老服务。市区农村和城镇低保标准统一提高到588元/人月，五县（市）农村低保标准提高到336元/人月以上。“中国最具幸福感城市”排名第一。社会事业加快建设。优质学前教育、高中教育覆盖率分别达到74.5%、84.4%。上城、建德等9个区、县（市）通过全国义务教育发展基本均衡县验收。解决进城务工人员子女入学23.75万名。新建改扩建幼儿园111所、省级示范职业学校4所，启用杭师大仓前校区（一期）。市属职业院校建设扎实推进。大力推进健康城市建设，滨江医院和下沙医院建成启用，96%以上基层医疗机构实现一体化管理。医疗诊间付费结算被评为全国十大最具影响力医改新举措。城乡爱国卫生运动继续在全国保持领先。浙江音乐学院加快建设，奥体博览中心主体结顶。世界文化大会、太湖文化论坛在杭举行。良渚古城遗址入选“世界十大田野考古发现”。群众文化体育事业稳步推进，竞技体育全国排名第三。民族宗教、红十字、妇女儿童、慈善等事业健康发展。社会管理取得实效。开展“全国和谐社区建设示范城市”创建工作，全市登记注册和备案社会组织共16615家，初步建立四级社会组织服务平台网络。推进网格化管理，完善大调解机制，成功调解各类矛盾纠纷8.6万件。构建防欠薪工作长效机制，劳动关系和谐指数连续3年全省第一。继续实施律师进社区进农村活动。全力做好禽流感、高温干旱和台风带来的洪涝灾害等防控工作，确保人民生命财产安全。食品药品重大安全事故零发生，地质灾害连续3年零死亡，安全生产主要事故指标连续10年负增长。加强社会治安综合治理，“平安杭州”建设取得新成效。全国纪念“枫桥经验”50周年现场会在杭召开。杭州“最美现象”先进经验报告会在北京人民大会堂举行。

　　（五）抓重点，建机制，环境保护稳步推进

　　生态建设有序开展。深入实施“美丽杭州”建设“九大行动”及51项重点任务。中国生态文明论坛在杭召开。余杭区、江干区分别通过国家级生态区验收和技术核查。新增国家级生态乡镇（街道）13个、市级生态（文明）村105个。建成“三江两岸”沿江生态景观带75公里，完成绿化150万平方米，累计建成绿道211公里。“四边三化”工作走在全省前列。城区新增绿化面积424万平方米。西湖长桥溪水生态修复工程荣获联合国人居署改善居住环境最佳范例奖。环境治理力度加大。“清水治污”全面启动，建设污水管网190.41公里，新增截污量3.14万吨/日。整治黑臭城市河道53.8公里。地表水市控以上断面达标率为87.2%。启动钱塘江原水异味应急处理机制，加大自来水深度处理力度，全力排查污染物质。制定实施大气重污染应急预案。启用加气站2座，汽油油品提升到国Ⅳ标准。主城区基本建成“无燃煤区”。持续开展垃圾清洁直运，垃圾分类工作国内领先。节能减排扎实推进。开展国家节能减排财政政策综合示范试点。推进“十城千辆”等工程，投入使用节能与新能源汽车2793辆，其中新能源汽车1674辆。整治制革、印染、造纸等高污染排放企业379家，其中关停造纸企业105家。完成减排项目326个，脱硝改造84台热电锅炉和15条水泥熟料生产线。淘汰黄标车31170辆，查处高污染车辆4352辆次。成立市排污权交易中心，13家企业开展排污权交易。单位GDP能耗预计下降3.5%。

　　（六）抓改革，提效能，政府建设不断加强

　　依法行政水平不断提高。认真落实市人大及其常委会决议决定，积极支持市政协履行政治协商、民主监督和参政议政职能。提交地方法规草案4件，制定和修改市政府规章5件。承办市人大代表建议620件、市政协提案677件，满意率分别为99.4%、99.6%。市人大8件重点建议、市政协3件全会建议案和8件重点提案全部按时办结。行政效能建设取得实效。加快“两集中、两到位”职能整合，审批职能归并率达100%。精简行政许可事项108项、非行政许可事项330项，减少51.2%。推进投资项目审批流程改革，加强中介机构管理，压缩审批时间30%。代办审批项目3863个，重点项目代办服务实现全覆盖。全面建成四级行政（便民）服务中心。“市民之家”受理各类事项276万件，满意率达99.99%。上城区“政府管理与公共服务标准化”项目获“中国地方政府创新奖”。廉政和作风建设扎实推进。扎实开展党的群众路线教育实践活动，大力整治“四风”问题。深化政府部门综合考评，开展“效能亮剑”、公述民评等活动。做好领导干部和国有企业负责人审计工作。落实“八项规定”，市级行政事业单位会议活动减少32.7%，文件减少18.5%，“三公”经费下降24.6%。开展对重点工程项目综合监督，节约资金2341万元。加大执法监察和执纪办案力度，避免和挽回经济损失1.8亿元。

　　过去一年，我们扎实推进国防、民兵预备役、人防、打私、防震减灾和机关事务工作，积极支持工会、共青团、妇联、侨联、残联、科协等开展工作。

　　各位代表，过去一年的成绩来之不易，这是党中央、国务院亲切关怀的结果，是省委省政府和市委正确领导的结果，是全市人民同心同德、共同奋斗的结果。在此，我代表市人民政府，向全市人民，向人大代表和政协委员，向各民主党派、工商联和无党派人士、各人民团体以及社会各界人士，向中央驻杭单位和省级部门、驻杭部队和武警官兵，向关心支持杭州建设发展的港澳台同胞、海外华人华侨和国际友人，表示衷心感谢并致以崇高的敬意！

　　在取得成绩的同时，我们清醒地认识到，杭州发展仍面临不少问题和困难。去年我市地区生产总值、社会消费品零售总额、节能、城镇和农村居民收入等指标低于预期目标。经济转型升级不快，创新驱动能力不足，缺少产业类大项目和战略性新兴产业支撑；空间、资源、环境刚性约束加大，副城组团承接能力不强，土地集约节约利用水平不高，节能减排压力加大；城市交通拥堵加剧，大气雾霾天数增加，河道水质改善不快，环境质量还不尽人意；就业、养老、教育、医疗等民生保障与人民群众的期望还存在较大差距；城乡区域发展还不平衡，低收入农户增收有待加快；城市防洪排涝设施建设滞后，应急救援能力有待加强；社会治理能力和城市管理精细化、科学化水平有待提高。

　　我们还清醒地看到，政府自身建设还有待加强，形式主义、官僚主义、享乐主义、奢靡之风还不同程度存在。少数干部存在骄傲自满、不思进取等思想。有的部门执行力不强，主动配合不够。部分工作人员作风不实，深入基层不多，联系群众不够，对群众反映强烈的问题解决不力。一些领域消极腐败现象仍然存在。对于这些问题，我们要高度重视，采取有力措施，切实加以解决。

　　二、2014年工作目标和总体思路

　　今年，世界经济将延续缓慢复苏态势，但不稳定不确定因素依然存在。我国经济将保持稳中有进、稳中向好态势，但也存在一些突出矛盾和问题。对杭州而言，既存在经济增速换挡、转型升级爬坡过坎、社会矛盾交织凸显等压力，也面临改革开放、新型城镇化、科技创新和市场化带来的难得机遇。为此，我们必须进一步坚定信心，拉高标杆，紧扣“杭改十条”，以勇立潮头、舍我其谁的气魄，以锲而不舍、昂扬向上的精神，以只争朝夕、敢为人先的闯劲，干在实处，走在前列，再创杭州发展新辉煌。

　　今年政府工作的总体思路是：全面贯彻党的十八大和十八届二中、三中全会精神及省委、市委决策部署，以科学发展观为统领，全面深化改革，加快转变经济发展方式，牢牢把握“稳中求进、改中求活、转中求好”工作总基调，稳增长、调结构、强统筹、治环境、惠民生、促和谐，保持经济持续健康发展和社会和谐稳定，继续在全省发挥龙头领跑示范带动作用，为建设东方品质之城幸福和谐杭州作出更大的贡献。

　　综合各方面因素， 2014年全市经济社会发展的主要预期目标为：地区生产总值增长8.5%左右；社会消费品零售总额增长13.5%，固定资产投资增长15%，外贸出口增长7.5%；地方财政收入增长8%；城镇居民人均可支配收入增长9%，农村居民人均纯收入增长10%；单位生产总值能耗、主要减排指标完成上级下达任务；居民消费价格涨幅控制在3.5%左右；城镇登记失业率控制在4%以内；人口自然增长率控制在6.8％。以内。

　　为实现上述预期目标，我们必须牢牢把握全局，突出重点，统筹兼顾，在新的起点上全面推进“改革创新年”、“项目推进年”、“优化服务年”，确保圆满完成全年各项工作任务。

　　——改革创新年，就是要解放思想，大胆探索，破除一切阻碍发展的体制机制障碍，正确处理政府、市场、社会三者关系，简政放权，充分发挥市场和社会的作用。要尊重群众的首创精神，鼓励创新，宽容失败，先试先行，大力推动理念创新、技术创新、管理创新、发展方式创新和体制机制创新，充分调动各层次各方面的积极性和创造性，放手让一切劳动、知识、技术、管理、资本的活力竞相迸发，让一切创造社会财富的源泉充分涌流，再造杭州高起点发展的新动力。

　　——项目推进年，就是要转变政府工作理念和工作方式，把改革创新、经济发展、城市建设、环境保护、社会事业、民生保障和社会管理等各项政府工作项目化，明确工作的路线图、时间表和责任制，做到工作项目化、项目目标化、目标责任化、责任考核化。抓铁有痕、踏石留印，以破竹之势狠抓落实，再现杭州高起点发展的新跨越。

　　——优化服务年，就是要转变职能、改进作风、优化服务、提高效率，营造更加规范高效的政务环境、公平诚信的市场环境、公开透明的法治环境、公正和谐的社会环境和秀美宜居的生活环境。通过服务基层、服务企业、服务群众，不断提升政府服务能力和服务水平，克服疲劳症，提振精气神，再创杭州高起点发展的新优势。

　　三、2014年重点工作

　　（一）坚持改革引领，着力激发杭州发展活力

　　全面深化重点领域和关键环节改革，推进政府治理体系和治理能力现代化建设，充分发挥市场在资源配置中的决定性作用，更好发挥政府作用，激发全社会创业创新活力。

　　加强政府自身改革。进一步强化政府公共服务、市场监管、社会管理和环境保护等职能，全面开展市、区（县、市）机构改革。积极推进大部门制改革，稳步推进事业单位分类整合和改革。加大权力下放力度，充分发挥区、县（市）作用。组建市场监管局，加强食品药品全程管理。建立市产业促进、城市国际化、生态文明建设等综合协调机构。进一步理顺萧山、余杭两区融入主城区的体制机制。积极推进市域行政区划调整。完善市区两级财政管理体制，促进城区基本公共服务均等化。激发市场主体活力。改革国有企业和国有资本经营体制，发展混合所有制经济，完善现代企业制度，增强国企创新能力和发展活力。鼓励民营企业和社会资本参与国企改革、基础设施和社会事业建设，大力推进民营经济强市建设。建立企业梯次培育制度，加大对大企业大集团、成长型企业、科技型小微企业扶持力度，鼓励企业兼并重组和上市。重视本地自主知识产权首台（套）推广应用。推广工商登记制度改革试点，建立“宽进严管”的市场主体信用监管体系。推进资源要素市场化配置改革。强化市场取向，制定要素市场化配置综合改革方案并开展试点，实施集约用地“亩产倍增”计划。探索建立城乡统一的建设用地市场，推进不动产统一登记。深化农村产权制度改革和集体资产股份制改革。规范发展民间融资，拓宽企业直接融资渠道。深化能源供应机制改革，开展区域用能权交易试点。完善用电、用气、用水差别化、阶梯式价格调节机制，实施企业初始排污权分配和有偿使用。建立亩产贡献、能耗、排污倒逼机制，加快淘汰落后产能。深化水资源配置改革，开展流域用水总量控制和水权制度改革试点。

　　（二）坚持创新驱动，着力转变经济发展方式

　　围绕“一基地四中心”建设，深入实施创新驱动战略，着力提升经济发展质量和效益，打造杭州经济升级版。

　　提高创新能力。全面落实“科技创新30条”，完善区域创新体系和科技创新激励机制，力争全社会研发经费占GDP比重达到3%以上。整合优化创新平台，发挥高新区（滨江）的创新引领作用，争创国家自主创新示范区。推动杭州经济技术开发区、大江东产业集聚区、青山湖科技城、未来科技城、钱江经济开发区等产业平台创新发展。深化与浙大、中国美院的战略合作，开展与中国工程院、北大合作，促进企业与在杭高校协同创新和产学研平台建设。健全公共创新三级服务体系，促进创业苗圃、孵化器、加速器健康发展。推广“创新服务券”，深化雏鹰计划、青蓝计划、蒲公英计划。重视企业家队伍建设，制定实施“创新人才推进三年行动计划”，建立产业紧缺人才预测预警机制，加强高技术和高技能人才引进培养。新增“国千”人选10名、“省千”人选40名、市“521计划”人选20名。加强知识产权保护，实施“八倍增、两提高”科技服务专项行动。创建全国质量强市示范城市。办好2014“创新中国”总决赛。大力发展新兴产业。聚焦十大产业，力争十大产业增加值增幅高于GDP增幅3个百分点，占GDP比重达48.5%。大力发展信息产业和信息消费。全面深化与阿里巴巴集团的战略合作，积极培育大数据、云计算、物联网、移动互联网等新兴业态。加快“智慧杭州”建设。实施“三网融合”，加快建设宽带和下一代互联网等信息高速公路。加强大数据应用，以“智慧民生”、“智慧政务”示范应用为突破口，扩大市民卡应用面，促进智慧产业和智慧服务发展。打造高技术产业基地，积极推动先进装备制造、3D打印、医疗健康、节能环保、新能源、新材料等新兴产业发展。举办中国电子商务博览会，加快国际电子商务中心和中国快递示范城市建设，促进电子商务和快递服务融合发展。大力发展动漫、游戏、影视、设计等优势产业，推动文创产业集聚集群发展，建设全国文化创意中心。加快钱江金融城和财富管理中心建设，发展私募金融、科技金融、互联网金融，打造区域性金融服务中心。深化国家服务业综合改革试点，积极培育高端生产性服务业和总部（楼宇）经济。提升传统优势产业。加快信息化与工业化、制造业与服务业深度融合，推动产品、品牌、组织和商业模式创新。加大龙头企业培育和产业集聚转型力度，提升传统产业竞争力。深入实施“四换三名”工程。实施“机器换人”三年行动计划，完成技改项目450个，投入资金450亿元，认定示范应用企业20家。淘汰水泥、造纸、化工、印染、铸造等高能耗企业130家，腾出用能指标20万吨标煤。盘活存量土地1.2万亩，消化批而未供土地5万亩。积极探索商业零售线上线下融合发展，力争网络零售额突破2000亿元。启动新一轮大企业培育五年行动计划。继续开展“个转企、小升规、规改股、股上市”工作。大力发展现代农业。完成粮食种植面积233万亩，健全粮食生产、流通、储备、调控体系，确保粮食安全。严格落实耕地保护，建设高标准基本农田26万亩。持续推进农业“两区”建设，新建10万亩粮食生产功能区，加快建设“六个百万”城市保供生产基地，“菜篮子”产品年直供销量达20万吨。实施现代农业“十大工程”，大力发展规模农业、高效生态农业、都市农业和设施农业。鼓励发展家庭农场、涉农网商、专业合作社等新型农业经营主体。

　　（三）坚持项目带动，着力促进经济平稳增长

　　充分发挥投资的拉动作用、消费的基础作用、出口的支撑作用，保持经济在合理区间平稳增长，实现杭州高起点上的新发展。

　　扩大有效投资。加大项目推进力度，力争全年实现固定资产投资5000亿元，其中民间投资突破2700亿元。加强项目引进和要素保障，确保招商引资规模、要素投入和投资额度的相互匹配。加大工业项目投入，积极开展工业技改“双百工程”，推进长安福特、东风裕隆、中科新松、杭汽轮重工等重大项目建设，完成工业投资1000亿元。加强新城和城市综合体建设，完成投资2188亿元。加快地铁、快速路网、高速公路、能源电力和城市排涝等重大基础设施建设，完成投资955亿元。继续实施优质教育、全民健康等“十大民生工程”，完成投资760亿元。促进消费增长。千方百计增加居民收入，认真落实促进消费各项政策措施。引导和鼓励大众消费，大力发展电子商务、品牌营销、连锁经营、物流配送等商贸业态。举办“购物天堂、美食之都”活动200场。推进旅游全域化，促进旅游与文化娱乐、体育休闲、健康养老、信息服务等业态融合发展，继续发展十大特色潜力行业。加快西博会市场化转型。发展“三江两岸”黄金旅游，培育农村生态旅游。完善延安路商贸功能，促进武林、湖滨、吴山三大商圈融合发展。加快国际商贸城建设，优化城区商贸中心和特色商业街区布局，鼓励发展社区和农村商业。推动全面开放。加强与上海自贸区对接，实施错位互补发展，推进跨境贸易电子商务服务试点。巩固传统国际市场，积极开拓新兴市场，改善外贸出口结构，支持自有品牌、自主知识产权和高新技术产品出口，完成市属出口413亿美元。大力发展服务外包，拓展生物医药、文化创意、研发设计、知识产权等服务外包新领域。支持企业“走出去”发展，培养本土跨国公司。加强与央企、500强企业和国际知名公司合作，加大对先进制造业、现代服务业和城市功能性项目的招商引资力度，引进外资60亿美元、内资1000亿元。持续做好“浙商回杭”工作，着力打造浙商总部中心。深化区域经济合作，推进与港澳台经贸、文化合作交流。

　　（四）坚持生态立市，着力加快建设美丽杭州

　　保护良好的生态环境，是全市人民的共同心愿和历史责任，必须把生态环境作为生命线来对待，宁可经济增长放慢一点，也要壮士断腕，下最大的决心、花最大的力气，打好环境治理攻坚战，保卫好我们共同的生活家园。

　　打好“五水共治”攻坚战。加快实施治水“三年行动计划”。在治污水上，全面推行“河长制”，落实责任制。加快大市政管网、泵站和五县（市）污水处理厂建设，实现市区雨污管网与道路建设同步。全面消除市区垃圾河，治理黑臭河，创建生态示范河道。深化农村河道清理，推进农村生活污水处理全覆盖。在排涝水上，改建、续建、新建五堡排灌站、三堡南排、钱江水利枢纽等防洪排涝工程。对易积水的住宅小区、铁路涵洞和道路进行排水改造，治理低洼积水点80个，实现重点区域、重点部位、重点路段“短时强降雨道路交通不中断”。在防洪水上，加固海塘建设，提高防洪（潮）设计标准为100年一遇。实施东苕溪防洪加固工程。除险加固小型水库27座和万方以上山塘159座。在保饮水上，强化水源保护区环境治理，加快实施九溪、赤山埠和祥符水厂技术改造，推进湘湖、三白潭等应急水源工程建设。启用闲林水库，开工建设千岛湖配水工程。在抓节水上，运用经济杠杆，推进生活和生产节水，建成节水型居民小区10个，万元GDP用水量降低5%以上。打好治气治废攻坚战。贯彻国务院《大气污染防治行动计划》，制定实施市大气污染防治实施细则。坚持区域综合防治与强化属地治理并重，加大大气污染防治力度，努力减少雾霾污染影响。继续开展对主城区重点燃煤企业改造，加快无燃煤区由主城区向萧山、余杭和五县（市）推进。加强机动车尾气排放达标管理，淘汰黄标车2万辆，严格限行黄标车。加大工业企业大气污染防治，推进挥发性有机物污染整治。提升城市垃圾处理能力，深入实施主城区垃圾清洁直运。开工建设九峰、萧山东片垃圾焚烧厂，扩建提标改造七格污水厂。加大对工业危险废物、医疗废弃物、污泥、餐厨垃圾处理力度。加强建筑垃圾、城市生活垃圾和农村秸秆等再利用。着力推进节能减排。深化国家节能减排财政政策综合试点，实行单位产出能源消耗强度和消费总量双控管理，开展单位能耗增加值贡献率排序并向社会公布。推进节能降耗十大工程。启动“智慧能源”建设，加强“万吨千家”企业（单位）节能监督。落实建设项目排污权总量控制、总量准入和削减替代制度，建立三级企业环境行为信用等级管理制度。加快推进半山、北大桥地区污染企业“清零行动”。加大印染、制革、造纸和化工行业整治力度。完善落后产能淘汰以奖代补机制。大力发展循环经济，推进重点节能工程，扩大节能产品应用。加大生态补偿、政府采购绿色环保产品、脱硝工程的工作力度。建立公安环保执法队伍，加大环保执法力度，做到执法必严、违法必究，使违法排放者付出惨痛的代价。深入实施生态保护。实施“美丽杭州”建设三年行动计划，推进绿色发展、循环发展、低碳发展，创建国家级生态市。严格控制“六条生态带”，推进“五区一园”建设。开展城市绿道系统、通风廊道等规划研究。加大“三江两岸”生态景观整治力度，深入开展“四边三化“行动，建设绿色廊道。加大农村环境整治和面源污染治理，打造美丽乡村升级版。继续推进国家级生态县市（区）、乡镇（街道）创建工作。支持淳安县建设省级重点生态功能区，打造“美丽杭州”建设实验区。

　　（五）坚持新型城镇化，着力推进城乡统筹发展

　　坚持以人为本，突出宜居宜业，优化城市空间格局，完善中心城市功能，加快副城组团和县城发展，加快城乡一体化步伐。

　　深入开展交通治堵。当前，正是杭城治堵的“关键期”、“阵痛期”，需要全市人民理解支持，共克时艰。全力实施交通治堵五年规划。加快城市主次干道建设，建成19条、续建15条、开工10条，打通“断头路”9条。打造“公交都市”，发展绿色交通。新增公交专用道50公里，提升完善BRT公交网络，加快建设地铁站公交直达驳运系统，提高公交分担率3个百分点。加快停车泊位建设，确保新增公共停车用地150亩。推进政企单位与相邻小区错时共享停车，推广社区大封闭综合管理。实施差别化停车收费，发展停车产业。实施精细化管理，建立交通组织方案第三方评估机制，科学实施“错峰限行”。加大交通信息化建设力度，推行科技治堵。完善政府购买公共交通服务，引导市民绿色低碳出行。提升中心城市功能。加快杭州都市圈建设，提升在长三角的优势地位。开展城市总体规划修改，实现与土地利用规划、产业发展规划、生态保护规划“四规合一”。科学划定主城区开发边界和生态红线，合理控制主城区人口规模，加快城市建设重心向副城、组团转移。做好西湖、运河、钱塘江沿岸“三线”专项规划。开展地下空间利用、排水防涝、保障性住房、养老设施、城市环境卫生、公共服务配套等专项规划修编。推动城市公共设施与基础设施建设标准国际化，大力引进国际（合作）学校和医院，推进国际化生活社区和商业街区建设，提升城市国际化水平。加大城市建设力度。坚持拥江发展，加快城市东扩步伐。继续加快“三副六组团”建设，推进城东新城、之江新城、运河新城和钱江科技城建设。加快大城北地区、望江地区城市有机更新。加快地铁建设，续建2号线、4号线和1号线延伸段，开工建设5号线和6号线。加快城市快速路网建设，续建和开建紫之隧道、东湖路、文一路下穿隧道等框架性城市道路，确保秋石快速路三期主线、紫金港隧道、余杭塘路建成通车。改建机场高速，加快建设杭长客运专线、杭新景高速（建德段）、沪杭高速（抬升）等对外快速通道。加快推进杭黄铁路、千黄高速（淳安段）、临金高速（国高网段）、绕城西复线等项目。开展入城口整治。深化市域轨道富阳线、临安线等前期研究。继续推进富春江船闸改造、京杭运河二通道等项目建设。提高城市管理水平。全面开展“贴心城管”行动，深入推进“智慧城管”建设，创新城市管理运行机制，努力提升城市管理水平。推进城市管理重心下移，鼓励社会公众参与城市管理，大力推进“门前新三包”。落实长效管理机制，重点开展对建筑渣土、查违控违等治理，实施城乡结合部垃圾中转站提升改造。加强供水、供电、供气等市政公用设施安全运行监管。以创建“无违建”县、乡镇（街道）为目标，大力推进城乡“三改一拆”，重点整治“一户多宅”。推进城乡融合发展。加大城乡统筹力度，深入实施科技、现代服务业、文创、旅游、交通和人才“六大西进行动”，推进产业向县（市）梯度转移，增强县城、中心镇产业承接能力和人口承载能力。深入实施“双千工程”、“大企业大集团服务中心镇建设”活动，加大社会资本参与中心镇建设支持力度。继续开展中心村、精品村、风情小镇、精品线路和精品区块建设，保护好文化遗存。深化“联乡结村”，持续实施低收入农户收入倍增计划。改善山区农民饮用水条件。做好农村住房改造工作。

　　（六）坚持以人为本，着力提高人民群众福祉

　　按照“保基本、兜底线、促公平、可持续”的总体要求，坚持政府新增财力三分之二以上用于民生保障，建立和完善可持续的城乡一体基本公共服务体系。

　　努力完善保障体系。促进高质量的充分就业，新增城镇就业22.6万人，新增大学生创业企业1000家。深化“春风行动”，做好低收入和生活困难群体的基本生活保障。建立健全食品药品安全责任体系和基层监督网络，争创国家食品安全示范城市。加快形成“9064”养老服务格局，提高社会办机构养老比重，提升社区养老服务。完善住房保障体系，开工建设保障性安居房30238套，竣工35778套。大力发展社会事业。推进学习型城市建设。深化名校集团化办学和城乡学校共同体建设，推进县（区）域内义务教育公办学校教师校长交流。继续实施名师公开课和阳光招生。支持杭师大转型发展，推进市属高校产教对接，加快现代职业教育体系建设。支持民办教育健康发展。加快推进市属公立医院改革。推广“智慧医疗”，实现医院检查检验结果互认、市民卡诊间结算向市直管民营医疗机构延伸。建成启用市妇女医院，推进市儿童医院（二期）和市中医院丁桥分院建设。提高基层医疗卫生服务能力。落实单独两孩政策。大力繁荣文化事业。推进文化名城建设，不断提升城市软实力。弘扬“最美现象”，倡导社会主义核心价值观。大力实施文化惠民工程，努力构建市域一体的公共文化服务体系。传承历史文脉，继续实施西湖、西溪、市区河道等综保工程，积极推进运河和良渚遗址申遗。加强对历史文化街区和城市特色风貌区保护，加大对“老字号”、古树和古建筑保护。加快之江文化大平台、动漫馆建设，提升国际动漫节、西博会、文创博览会等影响力。加快建设奥体博览城，积极申办大型国际国内赛事。合力加强社会治理。创新社会治理体制，鼓励和引导社会组织参与社会事务管理。深入开展网格化管理、组团式服务和综合式执法，共建共享“平安杭州”。健全社会普法机制，深入开展“平安网格”创建活动，创建“全国和谐社区建设示范城市”。发扬“枫桥经验”，完善社会矛盾大调解体系，有效化解企业改制、征地拆迁、医疗等领域的矛盾纠纷，把涉法涉诉信访纳入法治轨道处置。推进户籍制度改革，改进流动人口管理和服务，稳妥推进农业转移人口市民化。规范劳务派遣，构建和谐劳动关系。全面实施重大项目、重大决策社会稳定风险评估。加强社区矫正和安置帮教工作。加强安全生产和食品药品、消防等公共安全监管，健全突发事件应急处置机制。切实做好国防建设、国家安全、人民防空和双拥优抚工作。

　　（七）坚持为民惠民，着力办好十件实事

　　为民解忧是人民政府应尽的职责，今年将继续围绕人民群众关心的切身利益问题，全力办好十件实事。

　　（1）持续改善市民出行条件。新辟10条、优化30条公交线路，新增公共自行车3000辆，增扩服务点80处。新增1000辆双燃料出租车。开通试运营地铁2号线东南段。建成10处人行过街设施。新增5万个停车泊位。（2）加强雾霾治理。新增城市绿地300万平方米。淘汰主城区1136辆公交黄标车。有效控制市区建设工地扬尘，评选100个文明施工标准化工地。（3）提升饮用水质量。关停搬迁一、二级饮用水源保护区内污染企业20家。完成1万户老旧住宅“一户一表”安装，开展高层住宅二次供水试点。巩固提升99%以上农村集中式供水率。（4）提升城市河道水质。新增城镇污水管网100公里，完成3万吨/日污水截污纳管，消除排污口300个。治理47条黑臭河道，创建5条（段）生态示范河道。（5）加强食品药品安全监管。食品定量检测达4批次/千人，合格率达到95%，其中地产蔬菜、生猪等主要农产品合格率达到97%以上。确保药品抽验2000批次以上，快速检测1800次以上。学校食堂主副食品统一配送或定点采购率达90%以上。（6）提高社会保障水平。全市基本养老和医疗保险参保率分别达94%和98%以上。继续提高企业退休人员基本养老金。主城区居民基础养老金标准提高到150元/月，萧山、余杭区和五县（市）有所提高。持社会保障卡人数达760万。（7）推进养老助残服务。新增养老床位6000张。享受政府购买居家养老服务达到6万人。改扩建居家养老服务照料中心500家。实施0-6周岁残疾儿童抢救性康复项目。（8）丰富城乡文体生活。建成150座农村文化礼堂。减免4万户以上低保困难家庭有线电视入网费和收视服务费。新置10套24小时自助式微型图书馆、20处城乡公共体育设施。市区中小学校体育场地向社会开放率达90%以上。（9）优化小区居住环境。新增5万户管道燃气用户。新建2万座农村无害化厕所。改造40座城区公厕及配套服务设施。消除1000处城市照明暗区。主城区提升改造150处社区便民服务点。（10）建立便民E邮站。建立E邮站快递包裹智能存取服务平台。主城区建立800个“E邮柜公共服务”站点，提供24小时自助投取包裹服务。

　　（八）坚持依法行政，着力提升政府治理能力

　　围绕打造“为民、务实、清廉”人民满意政府的总要求，进一步转变政府职能，创新管理方式，提高管理效能，努力建设法治政府和服务型政府，不断提高政府的执行力和公信力。

　　依法履行政府职责。坚持依法治市、依法行政，全面贯彻实施《浙江省法治政府建设实施标准》。规范决策程序，提高民主决策、科学决策和依法决策水平。坚持政府重大决策向人大及其常委会报告制度。深化政府与政协年度工作计划协商制度。自觉接受人大法律监督和政协民主监督，主动接受司法监督、舆论监督和社会大众监督。加强政务信息公开，完善政府及政府部门新闻发言人制度。加强审计监督，健全政府债务风险管控机制。提高行政审批效率。加大简政放权力度，建立完善权力清单制度和负面清单制度，推进行政审批标准化建设。按照“减、放、提、管”思路，进一步精减审批事项，压缩审批时间，加大向基层放权分权力度，加强审批项目事中事后监管和对中介组织的管理。抓好国际商贸城行政审批权力下放试点。继续推行投资项目“五阶段”审批、工业园区“化零为整”审批。切实转变工作作风。深入开展党的群众路线教育实践活动，不断加强干部队伍建设。全面落实勤政廉政各项规定，加强对重点领域和重点环节监督，建立健全惩治和预防腐败体系。严格执行“八项规定”，抓好“四风”问题整改，严肃整治景区公园会所中的歪风。进一步厉行节约，精简会议和文件，规范公务接待，严控创建达标检查评比和节庆论坛等活动，全市“三公经费”支出下降30%，全部用于治水。完善“公述民评”、“效能投诉”等载体，强化社会评价意见整改。

　　各位代表！2014年工作的目标任务已经明确，前进的号角已经吹响，让我们紧密团结在以习近平同志为核心的党中央周围，在省委省政府和市委的坚强领导下，紧紧依靠全市人民的智慧和力量，解放思想、锐意进取、鼓足干劲、奋力拼搏，为打造东方品质之城、建设幸福和谐杭州而努力奋斗！
